# Targeted chromatin conformation analysis identifies novel distal neural enhancers of *ZEB2* in pluripotent stem cell differentiation

**DOI:** 10.1093/hmg/ddaa141

**Published:** 2020-07-06

**Authors:** Judith C Birkhoff, Rutger W W Brouwer, Petros Kolovos, Anne L Korporaal, Ana Bermejo-Santos, Ilias Boltsis, Karol Nowosad, Mirjam C G N van den Hout, Frank G Grosveld, Wilfred F J van IJcken, Danny Huylebroeck, Andrea Conidi

**Affiliations:** 1 Department of Cell Biology, Erasmus University Medical Center, Rotterdam, CN 3015, The Netherlands; 2 Center for Biomics, Erasmus University Medical Center, Rotterdam, CN 3015, The Netherlands; 3 Department of Molecular Biology and Genetics, Democritus University of Thrace, Alexandroupolis 68100, Greece; 4 Department of Biochemistry and Molecular Biology, Medical University of Lublin, Lublin 20-093, Poland; 5 Department of Development and Regeneration, KU Leuven, Leuven B-3000, Belgium

## Abstract

The transcription factor zinc finger E-box binding protein 2 (ZEB2) controls embryonic and adult cell fate decisions and cellular maturation in many stem/progenitor cell types. Defects in these processes in specific cell types underlie several aspects of Mowat–Wilson syndrome (MOWS), which is caused by *ZEB2* haplo-insufficiency. Human *ZEB2*, like mouse *Zeb2,* is located on chromosome 2 downstream of a ±3.5 Mb-long gene-desert, lacking any protein-coding gene. Using temporal targeted chromatin capture (T2C), we show major chromatin structural changes based on mapping in-*cis* proximities between the *ZEB2* promoter and this gene desert during neural differentiation of human-induced pluripotent stem cells, including at early neuroprogenitor cell (NPC)/rosette state, where *ZEB2* mRNA levels increase significantly. Combining T2C with histone-3 acetylation mapping, we identified three novel candidate enhancers about 500 kb upstream of the *ZEB2* transcription start site. Functional luciferase-based assays in heterologous cells and NPCs reveal co-operation between these three enhancers. This study is the first to document in*-cis* Regulatory Elements located in *ZEB2*’s gene desert. The results further show the usability of T2C for future studies of *ZEB2* REs in differentiation and maturation of multiple cell types and the molecular characterization of newly identified MOWS patients that lack mutations in *ZEB2* protein-coding exons.

## Introduction

Genome-wide identification of regulatory elements (REs), using the mapping of epigenetic modifications and transcription factor (TF) binding sites coupled to chromatin conformation capture analyses at different resolutions (3C and beyond), strongly indicate the occurrence of DNA looping between promoters and distal enhancers in controlling cell fate and differentiation. These transcriptionally functional DNA loops occur within large interaction-domains, the so-called topologically associating domains (TADs), which themselves are genome-structural loops connected by linker regions (1–5). The intra-TAD DNA loops formed by in-*cis* promoter-RE proximities lead to precise cell type/stage-specific regulation of gene expression (6–9). REs are often evolutionary highly conserved and usually flank genes shown to control development and cell differentiation, but also metabolic pathways (10). For example, in pluripotent stem cells (PSCs), structural ‘hubs’ can be observed wherein the core pluripotency factors Sox2, Nanog and Oct4 control genes involved in the maintenance of pluripotency (e.g. *Tcf3*, *Smarcad1*) (11). In these PSCs, the transcriptionally inactive regions are less organized, but these regions become more organized during differentiation to somatic cell types (11). It is also generally accepted that reprogramming of somatic cells, based on transduction of Oct4, Sox2, Klf4 and c-Myc (OSKM) expressable cDNA, results in an almost complete reorganization of the genome architecture, which then becomes similar to that of PSCs.

Zinc finger E-box binding protein 2 (Zeb2) is a TF critical for vertebrate embryogenesis, including the development of the central nervous system (CNS) and peripheral nervous system (PNS) (12,13). In differentiating mouse embryonic stem cells, Zeb2 is needed for the exit from primed pluripotency and general as well as neural differentiation (14). In oligodendrocyte precursor cells during embryonic CNS myelinogenesis and adult Schwann Cell function in PNS (re)myelination, Zeb2 plays a dual transcriptional regulatory role, i.e. directly repressing genes involved in inhibition of differentiation, while directly activating (other) genes promoting cell differentiation and maturation. In doing so, Zeb2 generates the necessary anti-bone morphogenetic protein (BMP)(-Smad) and anti-Wnt, and in the PNS also the anti-Notch and anti-Sox2 activities needed for normal progression of commitment, differentiation and maturation in this glial cell lineage (15–18). In humans, *ZEB2* haplo-insufficiency causes the rare Mowat–Wilson syndrome (MOWS, OMIM #235730). Patients exhibit severe intellectual disability, epilepsy and/or seizures, Hirschsprung disease, and other anomalies including typical craniofacial defects (19–21). Analysis of the spectrum of the *de novo* mutant *ZEB2* alleles in a more recent cohort of 87 patients indicated for the first time that the severity of MOWS may correlate with the type of mutation (21). *De novo* deletions in the *ZEB2* gene that involve protein-coding exons or cause protein C-terminal truncation due to mutation into a stop codon, as well as even larger genomic deletions, cause severe defects, whereas the few known missense mutations (1.5% of about 320 exon-sequenced MOWS patients thus far) present with a milder form of the syndrome (21,22).

Additional work done in mouse models, including rescuing conventional or conditional *Zeb2*^−/−^ knockout backgrounds via introduction of a *Zeb2* cDNA (provided as heterozygous or homozygous transgene), strongly suggests that proper control of Zeb2 amounts, including via steady-state mRNA levels, is critical for normal Zeb2 functions, as observed from the graded phenotypic severities of the *Zeb2* knockout and/or transgene combinations (23–26). In this respect, little is however known about the precise and temporal transcriptional control of *ZEB2*, and only few studies have thus far focused on candidate TFs that bind to the *ZEB2* proximal promoter (27,28). Recently, different enhancers were identified, mainly by documenting evolutionary conserved *ZEB2* containing and flanking regions, followed by validation in zebrafish, rodent models and/or *in vitro* cellular models. In addition, in a transgenic rat model, *Zeb2* is regulated in a tissue- and time-specific manner by an enhancer located 1.2 Mb upstream of the transcription start site (TSS) (29). In the subpallium of the developing mouse brain, two enhancers flanking the *Zeb2* locus have been proposed to be activated by the TF Dlx2 (30). More recently, combining publicly available databases of chromatin interaction and mapped histone signatures, again followed by validation in zebrafish, eight enhancers were identified in intergenic, intronic and exonic sequences of *ZEB2* (31). These enhancers are active in mid-/hindbrain regions, trigeminal ganglia, notochord or the whole brain (31).

Human *ZEB2* is located on chr2:145141942–145277958 (genome release GRCh37/hg19), downstream of a 3.3 Mb-long region lacking protein-coding sequences, which encodes several non-coding RNAs. The sequence of this region is locally highly conserved between different species, despite the differences in length of this region (chimp: 3.4 Mb; mouse: 3.7 Mb; chicken: 1 Mb; *Xenopus*: 1 Mb). This gene desert is located between *ZEB2* and *ACVR2A*, which encodes for the Activin type-IIA receptor, a component of the transforming growth factor type beta (TGFβ)/BMP signaling system (32). In a separate study, a map of non-coding elements involved in human cortical neurogenesis was obtained by combining chromatin accessibility and mRNA profiling data (33). Several non-protein coding elements are in proximity of *ZEB2*, including the long non-coding RNA (lncRNA) *LINC01412*, which maps roughly 2 kb upstream of the *ZEB2* TSS. In a genome-wide association study of more than 2400 cases of aortic valve stenosis (a pathology that hits about 5% of MOWS patients), this region harbors single-nucleotide polymorphisms in the non-coding RNA *TEX41*, located about 150 kb upstream of the *ZEB2* TSS, that directly interact with *LINC01412* and the *ZEB2* proximal promoter region (21,34). A schematic overview of the *ZEB2* locus, with published enhancers and chromatin interactions that co-regulate *ZEB2*, is depicted in Supplementary Material, Figure S1.

Altogether, and also considering its listing as a super-enhancer top-gene (35,36), the gene desert upstream of the human *ZEB2* becomes a priority for identifying candidate and/or pathologic *ZEB2* REs. Given the critical role of Zeb2 during exit from primed pluripotency, its dynamic regulation during neural and general differentiation of mouse embryonic stem cells, and applied rescues in knockout stem cells with inserted Zeb2 expressible cDNA (14), we decided to study chromatin conformation dynamics of the human *ZEB2* locus during neural differentiation of induced PSCs (iPSCs).

Several chromosome conformation capture techniques (3C, 4C, 5C, Hi-C, ChIA-PET) have been developed to investigate and characterize spatial genomic organization by chromatin interactions (37–41). These techniques are mostly expensive, require extensive primer design and have a resolution of tens of kb. Recently, targeted chromatin capture (T2C) was shown to virtually provide high resolution (in the order of few kb or even <1 kb) and combine this with high coverage and low sequencing efforts, hence at a contained cost (42). We therefore aimed to study the chromatin dynamics of the *ZEB2* locus, during iPSC neural differentiation, considering the whole gene desert and both its flanking regions, for a total genomic region of 7.4 Mb in length (coordinates chr2: 143270465–150642631; GRCh37/hg19 genome reference). Supplementary Material, Figure S1B shows this region of interest used in this T2C study.

By correlating chromatin architecture reconstruction via T2C at an average resolution below 1 kb with H3K27ac marks, RNA-profiling at selected time points of cellular neural differentiation, and further taking into consideration locus sequence conservation in vertebrates, we identified three novel candidate *ZEB2* enhancers. Our work demonstrates, for the first time, the dynamic regulation of *ZEB2* expression by distal REs that loop to the *ZEB2* promoter during cell differentiation. These studies are expected to open the road to improved and/or expanded genetic and additional functional characterization of those MOWS patients for whom no mutation affecting the protein-encoding sequence of *ZEB2* can be identified.

## Results

### Transcriptomic profile of neural differentiating human iPSCs

**Figure 1:**
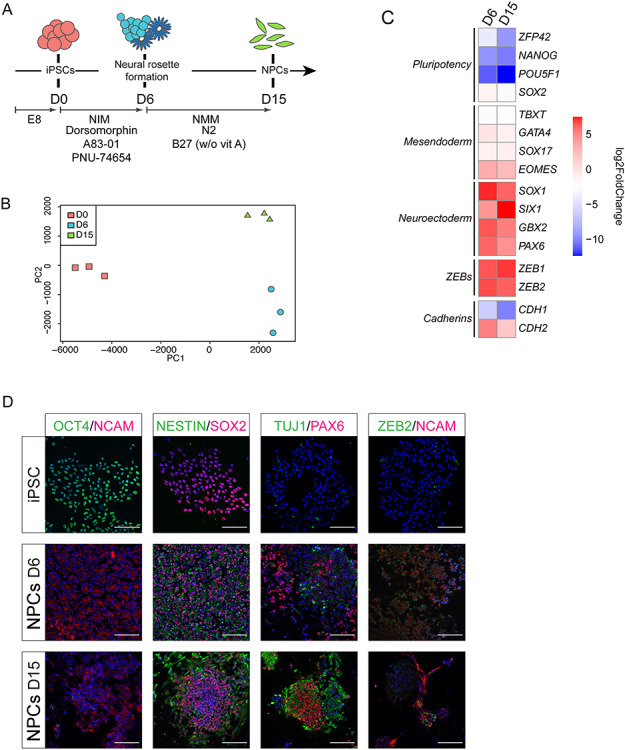
Gene expression profiling of differentiating iPSCs. (**A**) Schematic overview of the differentiation protocol used, including specific media and inhibitors (see Materials and Methods). Abbreviations used: EB, embryoid body; NIM, neural induction medium; D (day) 0, undifferentiated state; D6, early NPC/neural rosette formation; D15, NPCs; w/o vit A, no vitamin A added to B27. (**B**) Principal component analysis plot showing clustering of biological repeat (*n* = 3) RNA-seq samples based on time of differentiation. (**C**) log2Fold Change heatmap of selected marker genes confirming progression from pluripotency to efficient neural differentiation. (**D**) Immunofluorescence staining at selected timepoints of differentiation for pluripotency (OCT4, SOX2) and NPC markers (NCAM, NESTIN, TUJ1 and PAX6) and ZEB2. Scale bar = 100 μm.

RNA-sequencing of undifferentiated cells (D0), early neuroprogenitor cells (NPCs)/neural rosettes (D6) and late NPCs (D15) was carried out (Fig. 1A; see also Materials and Methods). Principal component analysis shows the clustering of the samples based mainly on the time point of differentiation (PC1, Fig. 1B). *ZEB2* mRNA, as well as the transcripts of the second ZEB-family member *ZEB1*, is upregulated already at D6 of differentiation (Fig. 1C; Supplementary Material, Fig. S2A). The acknowledged ZEB1/2 direct target gene *CDH1*, encoding for the epithelial cell specific homotypic cell-cell adhesion protein E-cadherin, was concomitantly downregulated already at D6 (Fig. 1; Supplementary Material, Fig. S2A). Its expression inversely correlated in the bulk cell cultures with that of the N-cadherin encoding gene *CDH2* (Fig. 1C; Supplementary Material, Fig. S2A).

Expression of genes encoding the core components of the pluripotency network, such as *NANOG*, *POU5F1* (*OCT4*), *SOX2,* and *ZFP42* (*REX1*), is downregulated upon differentiation (Fig. 1C; Supplementary Material, Fig. S2B). *SOX2* is however also critical to neurogenesis, and its expression—even though decreased during differentiation—remains high when compared to other pluripotency genes (43) (Supplementary Material, Fig. S2B). Conversely, the expression of neuroectoderm marker genes, such as *GBX2*, *PAX6*, *SIX1* and *SOX1* increased (Fig. 1C; Supplementary Material, Fig. S2C). Expression of mesendodermal genes *EOMES*, *GATA4*, *SOX17* and *TBXT* (*T*, *BRACHYURY*) was in this cell culture protocol not significant (Supplementary Material, Fig. S2D). Of the mesendodermal markers, only *EOMES* is upregulated at D6 and D15 compared to D0, most likely due to its proposed role in neurodevelopment (44,45). We have also performed staining for pluripotency marker proteins OCT4 and SOX2, for NPC markers NESTIN, TUJ1, PAX6 and NCAM, and for ZEB2, at the considered time points of differentiation (Fig. 1D). Taken together, the neural differentiation protocol of the initial human iPSCs was highly efficient.

### Chromatin dynamics of *ZEB2* locus during neural differentiation

**Figure 2:**
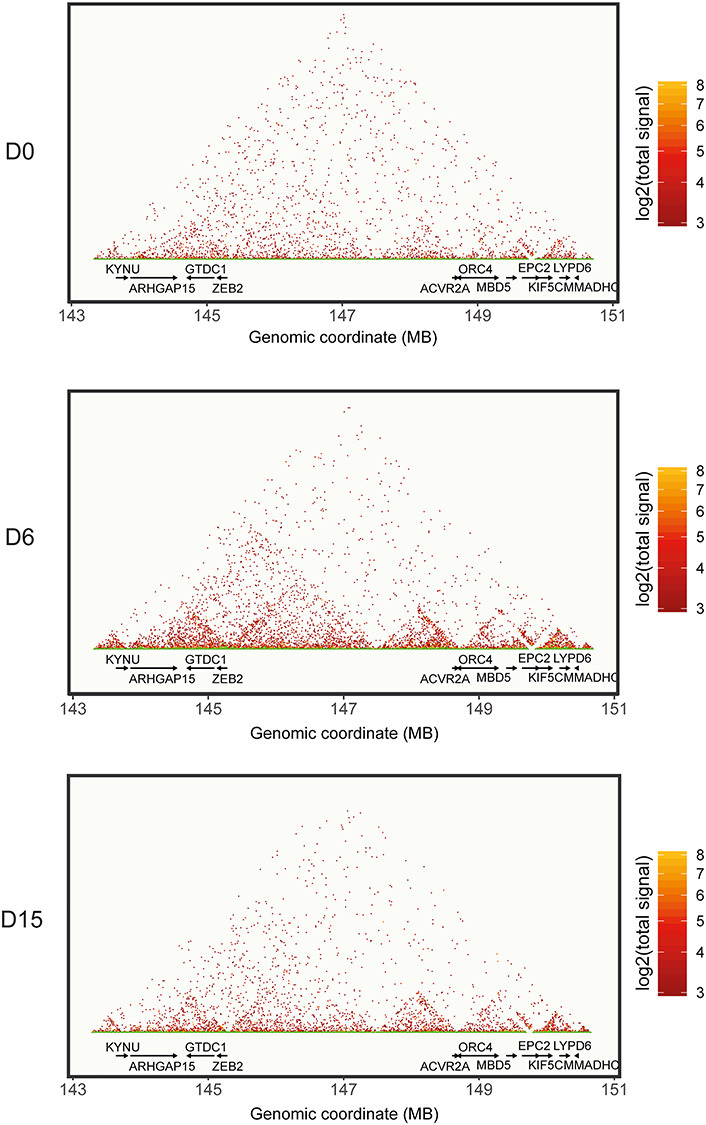
*ZEB2* locus dynamics during neural differentiation. Reconstruction of in-*cis* proximity interactions on the human chr2:143:151 region, including the *ZEB2* locus and the other annotated genes, in pluripotent (top panel, D0) and neural differentiating (D6, D15) iPSCs. To improve graphical clarity, the signals were binned to get a resolution of 20 kb.

We studied the regulation of the *ZEB2* locus by temporal T2C using the iPSC system at times documented above. First, we calculated the average size and density distribution of the fragments generated by *Apo*I cut in the considered area. Density distribution and frequency of the fragment size plots show a mean fragment size (dashed line in each panel of Supplementary Material, Fig. S3A) of ~500 bp *Apo*I, which did not change during differentiation. Reconstruction of high-resolution T2C maps at the different time points, plotting the single fragments obtained from the digestion with *Apo*I, resulted in a very sparse and unclear figure (data not shown). Therefore, we opted for reducing the resolution of our T2C maps by binning the signals to a resolution of 20 kb, resulting in an easier graphical interpretation (Fig. 2). On the other hand, the *Apo*I fragment resolution becomes very useful when zooming in on relatively small regions such as these shown in Supplementary Material, Fig. S4, depicting the *Apo*I fragment proximity interactions on the *ZEB2* gene *per se* (chr2:145141942–145277958, hg19) (see also Supplementary Material, File S1).

**Figure 3:**
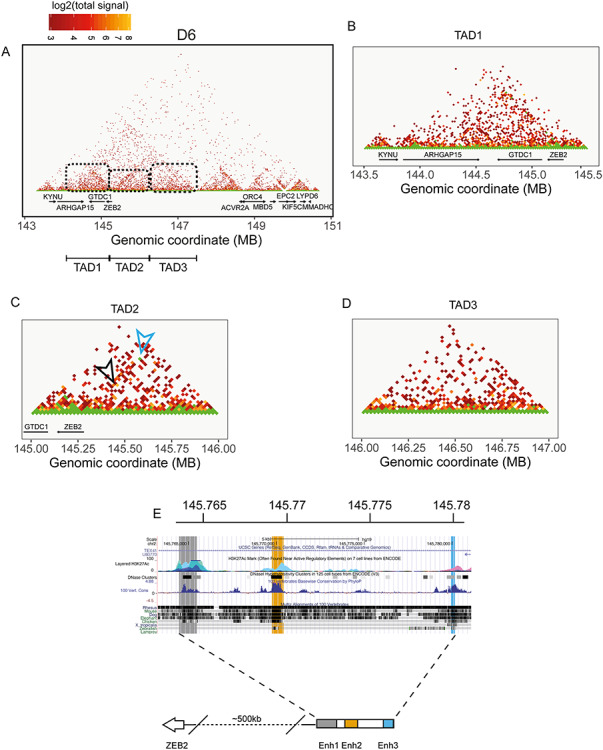
The main 143–147 Mb TAD defined at D6 is composed of three sub-TADs. At D6 (**A**), the major chromatin conformation encompassing the *ZEB2* locus and upstream region is evident, displaying more intragenic proximity interactions and also three well-defined TADs located between *ARHGAP15* and *ZEB2* (TAD1, **B**), between 145 and 146 Mb (TAD2, **C**) and a third one located in the *ZEB2* upstream gene desert between 146 and 147 Mb. At D6, the strongest signal is observed in a TAD structure defined by region coordinates 145260000–145280000 to 145760000–145780000 (**D**). Zooming-in on the coordinates chr2:145760000–145780000 allows the definition of three possible well conserved enhancers defined by H3K27Ac (**E**).

As shown in Figure 2, undifferentiated iPSCs revealed a diffuse pattern of proximity interactions with few appreciable TADs downstream of *ZEB2*, as well as along the gene desert and around the *ACVR2A* locus. At D6 (early NPCs), the TADs became more pronounced, with a long ~4 Mb TAD (chr2:143–147 Mb) being strongly defined and encompassing at least three sub-TADs. One, which we define as TAD1 (Figs 2 and 3B), bridged *ZEB2* to *ARHGAP15*; another (named TAD2) did so between *ZEB2* and a region located upstream its TSS in the gene desert (i.e. around chr2:146 Mb) (Figs 2 and 3C); and one (TAD3) between chr2:146 and chr2:147 Mb (Figs 2 and 3D). The major chromatin conformation change observed at D6 was concomitant with high *ZEB2* mRNA in early NPCs, while the less pronounced TADs at undifferentiated state were associated with low *ZEB2* expression (Figs 1 and 2). At D15 (NPCs), the 4-Mb TAD seemed less defined, whereas the three sub-TADs were still defined, even though their proximity signals were reduced, suggesting a slight loosening of the chromatin architecture (Fig. 2).

We also propose that several other TADs relate to the other genes, which are located in the same broad chromosomal region (*KYNU*, *ARHGAP15*, *GTDC1*, *ACVR2A*, *ORC4*, *MBD5*, *EPC2*, *KIF5C*, *LYPD6B,* and *MMADHC*) (Fig. 2). We therefore checked in our RNA-seq data whether transcription of these genes correlated with the formation of these TADs in the gene desert. In general, expression of these genes did not change during neural differentiation (Supplementary Material, Fig. S5A).

The sub-TAD TAD1 (chr2:144–145) bridges *ZEB2* with *ARHGAP15*, which encodes for a Rho-GTPase-activating protein, and is known to direct expression in both excitatory and inhibitory neurons of the adult hippocampus and midbrain (46,47) (Human Protein Atlas; https://www.proteinatlas.org) (Fig. 2). *ARHGAP15* mRNA expression was slightly upregulated at D6 in our RNA-seq data (Supplementary Material, Fig. S5A). Figure 3B and C show that *ZEB2* sequences are not only in proximity with *ARHGAP15*, but also that, at D6, *ZEB2* bridged with the 5′-region of *GTDC1*. According to the Human Protein Atlas database, the highest levels of GTDC1 are observed in the cerebral cortex (47).

Figure 3A and C, and Supplementary Material, Figure S4, also clearly show a local DNA looping of the *ZEB2* gene itself, which formed at D6 and then disappeared at D15, supporting therefore the results from Bar-Yaacov and co-workers (31) who identified *ZEB2* intragenic enhancers. *ACVR2A*, which flanks the gene desert, did not show a significant change in expression, even though TADs were forming and involved its coding sequence (Fig. 2A). *ACVR2A* is highly expressed in skin and skeletal muscle rather than brain regions, where it does not show any significant regional expression (47) (the Human Protein Atlas).

### T2C mapping and H3K27Ac marks identify three novel candidate enhancers for *ZEB2*

When zooming-in at D6 on the sub-TAD TAD2, formed by *ZEB2* and chr2:146, we noticed long-range proximity defining a sub-TAD that bridges ~chr2:145260000 (*ZEB2*) to ~chr2:145780000 (upstream gene desert) (blue arrow in Fig. 3C; Supplementary Material, File S2). The coordinates for the mapped proximity are chr2:145260000–145280000 (region A) and chr2:145760000–145780000 (region B). In the hg19 release, region A corresponds to the first 20 kb of *ZEB2*, including the promoter and (the non-coding) exon1 and (protein-encoding) exon2, while region B is located in an intron of the lncRNA *TEX41*, in the upstream gene desert (Fig. 3C and E). Another strong interaction signal forming a loop of 295 kb was also present (with coordinates chr2:145305000–145310000 and chr2:145600000–145605000) in the same area and formed another small TAD (black arrow, Fig. 3C). The coordinates for this proximity fall in one intron of *LINC01412* and one intron of *TEX41*. Both *LINC01412* and *TEX41* are very low expressed during neural differentiation of iPSCs (Supplementary Material, Fig. S5B). H3K27Ac marks are not present for this 295 kb proximity interaction (data not shown), whereas they are at the border of the sub-TAD formed by region A and B (Fig. 3E).

We therefore focused on the possible REs between region A and B that are associated with active enhancer marks (Fig. 3E). Combining H3K27ac marks and conservation tracks, region B can be divided in three clusters that possibly represent three novel candidate enhancers, named Enh1 (chr2:145764483–145765504), Enh2 (chr2:145769677–145770210) and Enh3 (chr2:145779965–145780193) (Fig. 3E). Taken together, these data suggest a time-regulated DNA looping with the aim of bringing the three enhancers and the *ZEB2* promoter in close proximity. Furthermore, this looping might specifically regulate *ZEB2* promoter, and gene transcription, during neural differentiation.

### The three novel candidate enhancers act on *ZEB2* promoter-based transcription

**Figure 4:**
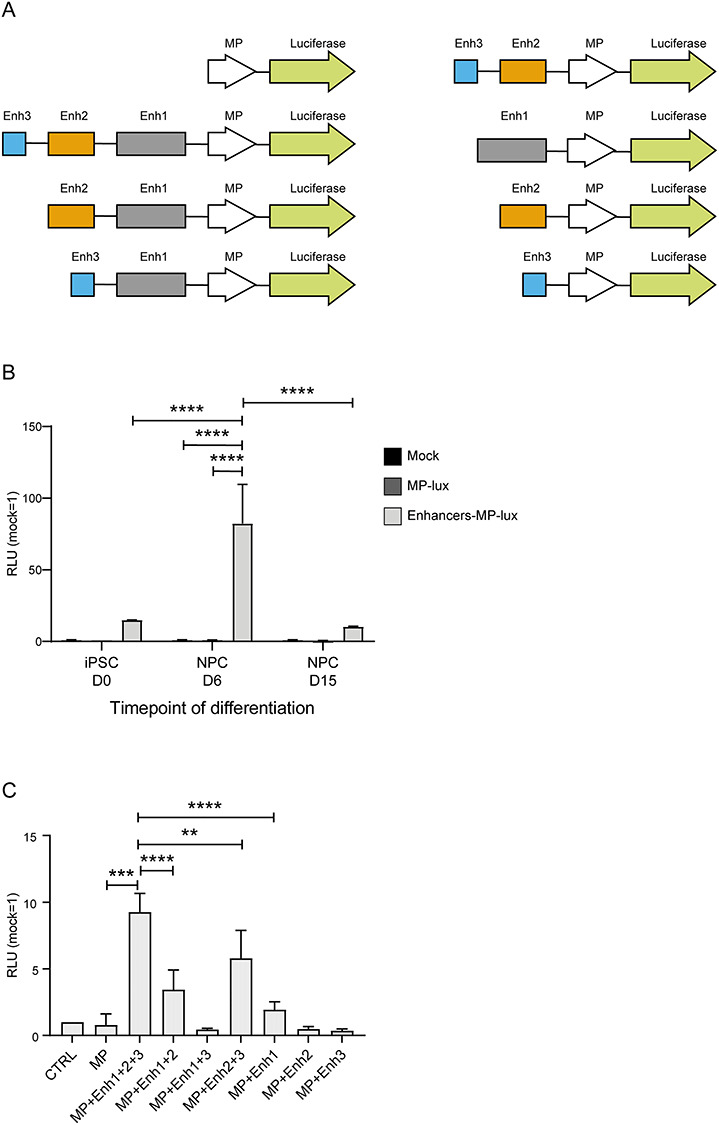
The three novel identified enhancers co-operate to drive upregulated activity of the minimal *ZEB2* promoter in a cell state/time specific manner. (**A**) schematic overview of combinatorial luciferase reporter-based constructs used in this study. (**B**) Luciferase assay performed in differentiating iPSCs transfected with a luciferase-based construct containing the three identified enhancers together with the minimal promoter of ZEB2 shows differential luciferase activation according the cell state A basal level of activation can be observed already at undifferentiated, D0, state. Similar level is also detected at mature NPC state, D15, while the highest expression is at D6, neural rosette/early NPC state. (**C**) Luciferase reporter assay of the whole panel of constructs, transiently transfected to heterologous HEK293T cells (see also the Discussion section). As for NPCs, the three enhancers positively co-operate. MP, minimal promoter of ZEB2. Error bars represent standard deviation of three independent biological replicates. Statistical significance was calculated with GraphPad Prism using a multiple comparison one-way Anova test. Asterisks represent *P*-values: ^*^P < 0.05; ^*^^*^P < 0.005; ^*^^*^^*^P < 0.0005; ^*^^*^^*^^*^P < 0.0001.

To functionally investigate the candidate enhancer regions identified by T2C in iPSCs at D6 of neural differentiation, we cloned these enhancers in combination with the *ZEB2* proximal promoter (chr2:145277927–145278000) in a luciferase-reporter-based vector and transfected these respective constructs in iPSCs at the different time points of differentiation (Fig. 4A and B). A similar basal level of luciferase activation is seen both at D0 (undifferentiated cells) and at D15 (mature NPCs), whereas at D6, the activation peaks to about 8-fold higher values (Fig. 4B), indicating that the three enhancers, tested away from their normal location, are bound by one or more transcriptional regulators specifically produced and active at this cell state.

We also produced combinatorial versions of the enhancers (Fig. 4A) and transfected the entire panel of enhancers-promoter combinations to heterologous HEK293T cells (Fig. 4C). The presence of all three enhancers and the promoter had the strongest effect on the vector-based luciferase activity. Enh2 in combination with either Enh1 or Enh3 also induced luciferase activity, albeit at a lower level. Remarkably, Enh2 by itself was not able to induce luciferase, indicating a co-operative effect of the three enhancers with Enh2, which enhances the stimulatory effect of the other two enhancers. The activity of Enh1 and Enh3 appeared to be additive, but only if Enh2 was present. We conclude that the three enhancers co-operate, including in the neural lineage, in driving *ZEB2* expression, and Enh2 + Enh3 are required for sustained *ZEB2* transcription.

### *In-silico* motif analysis predicts novel, remote-acting TF candidates for *ZEB2* transcription regulation

**Figure 5:**
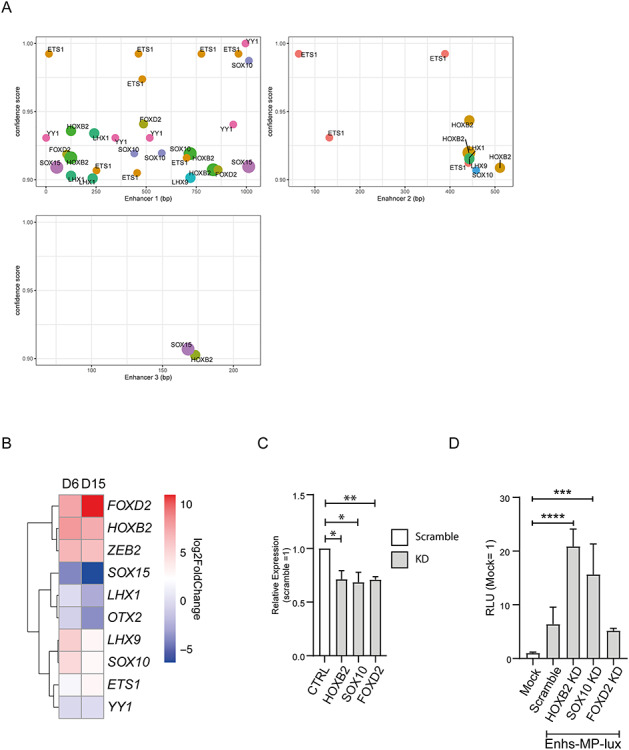
HOXB2 and SOX10 can bind the newly identified enhancers to regulate the ZEB2 minimal promoter activity. (**A**) localization of TF motifs in the sequence of the three enhancers using a confidence score >90%. (**B**) Differential expression, during iPSCs neural differentiation, of TFs for which a binding motif has been found in the enhancer sequences. FOXD2, HOXB2 are upregulated at D6 and D15 and might represent potential candidates able to bind the enhancers. (**C**) mRNA levels of HOXB2, SOX10 and FOXD2 after shRNA mediated KD in NPCs. (**D**) KD of HOXB2 and SOX10, but not of FOXD2, results in increased luciferase activation mediated by the three novel enhancers and the ZEB2 minimal promoter. Error bars represent standard deviation of three independent biological replicate. Statistical significance was calculated with GraphPad Prism using a multiple comparison one-way Anova test. Asterisks represent *P*-values: ^*^^*^^*^P < 0.0005; ^*^^*^^*^^*^P < 0.0001.

Next, we performed an *in-silico* prediction analysis of TF-binding elements present in the three enhancers. We used JASPAR database for human TF motif profiles, considering a >90% confidence score (48) (http://jaspar.genereg.net). Among the many and different motifs defined as such, we found collective enrichment for ETS1, FOXD2, HOXB2, LHX1 and 9, OTX2, SOX10 and 15, and YY1 in the enhancers (Fig. 5A). In particular, ETS1 seems a candidate for binding to Enh1 and Enh2, whereas Enh3 has just SOX15 and HOXB2 passing the applied 90% confidence threshold. Figure 5B shows the log2 Fold Change (log2FC) and Supplementary Material, Figure S6 the normalized values of the mRNAs for these TFs, as determined via our RNA-seq. *FOXD2* and *HOXB2* are the top upregulated genes among the possible TFs involved, while *SOX10* and *ETS1* show a very moderate upregulation (Fig. 5B). The other possible TFs are overall downregulated during differentiation (Fig. 5A).

To verify that FOXD2 and/or HOXB2 regulate the enhancers’ activity, we transfected NPCs with shRNAs directed against these two TFs and also for *SOX10*, for which a crosstalk with *ZEB2* is already known (49–53) (Fig. 5C). The knockdown (KD) of HOXB2 and SOX10 resulted in increased luciferase activity, while FOXD2 KD has no significant effect on the enhancers. Therefore, we propose that upstream HOXB2 and SOX10 might be necessary for or contributing to transcriptional repression of *ZEB2*.

## Discussion

Highly dynamic chromatin architecture accompanies stem cell differentiation, each change being reflected in state/stage-specific gene-signatures, often of direct relevance to specific lineage commitment and progression. Many genes involved in developmental processes that need to be temporally and/or spatially regulated are located in*-cis* of long gene deserts, such as *Shh*, *HoxD* and *Sox9* (54–56). These gene deserts contain several REs, either enhancers or silencers, which in a number of cases have been found to regulate the expression of the aforementioned gene(s) in a time, cell-type and/or tissue and location specific manner (54,57,58).

Here, we have started to add *ZEB2* as another acknowledged and developmental/disease relevant locus located downstream of a 3.3 Mb-long gene desert, with at least in the *ZEB2*-proximal 500 kb the identification of three hitherto unknown enhancers that co-operate in neural differentiation. These results demonstrate for the first time the value of T2C for studies of locus-specific transcription in the context of chromatin conformation and concomitant DNA looping. T2C has already been used to study global chromatin conformation and interactome at high-resolution (sub-kbp) and high coverage, with low sequencing efforts and at affordable cost (42,59–61). In addition, for *ZEB2* itself, functional studies and dynamics in cell differentiation (including in vertebrate models, but also in MOWS patients) T2C and hence DNA looping in the *ZEB2* locus can now be added to other types of functional study, including identification of ZEB2 direct target genes (ChIP-sequencing) and co-operation with other partners (ZEB2 interactome) (14,16,62–64). We have previously shown, both *in vivo* (often in the mouse) and *in vitro*, that detectable *Zeb2* temporal expression directly correlates with cell state and behavior (e.g. differentiation, maturation, migration, epithelial-mesenchymal transition) (23–26). In addition, by varying the dosage of *Zeb2,* in (rescued) knockout mice or via Zeb2 transgene-based (over)production in wild-type mice, the concept of precise dosage has become relevant to normal Zeb2 needs or functions, not only in developmental defect, but also pathology (49,51,65–67). Hence, studies of mechanisms that regulate *ZEB2* mRNA levels, as well as still needed studies documenting miR-based ZEB2 control and ZEB2 protein (in)stability, become increasingly relevant to the field.

In this study, for the first time for the *ZEB2* locus, we report genomic architecture dynamics and identify three novel enhancers, located about 500 kb upstream of the *ZEB2* TSS, regulating transcription of *ZEB2* during neural differentiation. We initially assess the expression profile of our iPSC line subjected to neural differentiation, and show that *ZEB2* is highly expressed at D6, corresponding to early NPCs. At this state, iPSCs have silenced almost completely their pluripotency gene signature and activated lineage-specific markers. This is in line with the observation by Chng and co-workers, who studied neuroectodermal differentiation of human ESCs, where double inhibition of Activin and BMP signaling results in increase of *ZEB2* mRNA levels up to 6 days of differentiation (68). In mouse ESCs, the levels of *Zeb2* mRNA rise at early NPC stage, after which they remain high (14). Similar to these ESCs, *ZEB2* expression is still sustained at NPC state as obtained in human iPSCs, and their NPC state is amenable to T2C analysis.

For our target region of interest, we designed probes to span roughly 7.4 Mb of chr2, i.e. 143270465–150642631 (hg19 genome reference). These probes cover *KYNU*, *GTDC1*, *ZEB2*, *ACVR2A*, *ORC4*, *MBD5*, *EPC2*, *KIF5C*, *LYPD6B,* and *MMADHC* protein-encoding genes, as well as the two, rather long lncRNA genes *LINC01412* and *TEX41*, located about 2.6 kb and 160 kb upstream of the *ZEB2* TSS, respectively. Our reconstruction of the in-*cis* dynamics of this region of chr2 shows that at iPSC state, the majority of the detectable proximities are short-range without a clear TAD structure, indicating a closed conformation in which the *ZEB2* gene is tightly packed and not accessible. As neural differentiation proceeds, the chromatin organization reveals well-defined, distinct TADs, and hence several proximities are mapped, and *ZEB2* is significantly upregulated. The main sub-TAD involves a loop between the *ZEB2* promoter and a segment of ~500 kb upstream of its TSS. Based on histone-3 marks and evolutionary conservation, we identify via T2C three REs, i.e. Enh1, Enh2 and Enh3, which are active enhancers. Previous studies, nearly exclusively done using computational analysis of publicly available databases, have shown the existence of different enhancers located upstream, downstream and intergenic/intronic to *Zeb2/ZEB2* (30,31). McKinsey and co-workers propose for their mouse models that two *Zeb2* REs are regulated by Dlx1/2, more specifically in gamma-aminobutyric acid (GABAergic) interneurons of the embryonic ventral forebrain (30). One of these REs is an enhancer located about 1.4 Mb upstream of the *Zeb2* TSS. Bar-Yaacov and co-workers identified eight new possible brain-specific human enhancers, albeit with intergenic or downstream location with regard to *ZEB2* (31). In our T2C approach, we also observe, at early NPC state, a major chromatin conformational change in the region containing *ZEB2,* corroborating the finding by Bar-Yaacov and colleagues on the regulation of *ZEB2* by intragenic enhancers.

The test of our three novel DNA looping segments for enhancer activity after transient transfection at each considered timepoint of differentiation yields that, concomitantly with the major chromatin remodeling observed at D6, the highest levels of the luciferase reporter are observed at the same timepoint/cell state, while a basal, comparable activation is seen at D0 (undifferentiated state) and D15 (late NPCs). Transfection of a series of combinatorial enhancers indicates that the three enhancers work synergistically and that Enh2 and Enh3 alone do not exert the same effect as Enh1 does, but they seem to act together. On the basis of the relative roles for Enh1 and Enh3, we propose that these two enhancers co-operate in a time and/or tissue specific manner, but only when enhancer 2 is present. Of the many TFs that can potentially bind these novel enhancers, HOXB2 and SOX10 are interesting candidates. While SOX10 has been demonstrated to associate as a protein with ZEB2 (52,53), nothing is known about a possible interaction between ZEB2 and HOXB2. KD of HOXB2 and SOX10 and assessment of the luciferase reporter activity in NPCs reveal a role for both TFs as transcriptional repressor of *ZEB2*; lower levels of HOXB2 or SOX10 result in increased expression of the luciferase reporter. HOXB2 is crucial for proper hindbrain formation and regulation of oligodendrogenesis in mice, both processes involving the proper formation of rhombomere 3 (69,70). Hindbrain-specific enhancers of *ZEB* have been identified by Bar-Yaacov and co-workers, suggesting that disruption of these enhancers might affect *ZEB2* expression in the hindbrain and ultimately proper hindbrain organization (31). In mice, in oligodendrocyte precursor cell Hoxb2 activates *Olig2* transcription, which is critical as upstream activating TF for *Zeb2* (15). Once the levels of Zeb2, as a result of *Zeb2* mRNA upregulation, are sufficiently high, Zeb2 TF acts in a dual mode: it activates genes promoting myelinogenesis in the embryonic CNS, whereas it represses other acknowledged genes that inhibit it (15). We add yet another potential mechanism underlying precise *ZEB2* transcriptional control and propose that HOXB2 may be needed for or contribute to repression of the newly identified enhancers and therefore also subsequent precise human *ZEB2* activation and/or upregulation.

In addition to the growing number of control mechanisms for this critical gene in various stages of development, adult homeostasis, and differentiation and/or maturation of many cell types, this will prompt the field to continue to document similarities or differences between human and e.g. mouse models, further assess these controls both *in vivo* and *in vitro*, for example also involving NPCs, and study fine-tuning of *ZEB2* mRNA levels at multiple levels, including locus-specific chromatin conformational changes. Here we find that the major impact of our newly identified enhancers is to have *ZEB2* expression peak at D6 of differentiation, whereas at D15 (late NPCs) its steady-state transcription is again lower. The chromatin conformation change we observed at D6 might be required for a transcriptional boost to increase *ZEB2* mRNA levels, such that they become sufficient for the cells to further proceed with differentiation. At D15, TFs, such as HOXB2, which are produced in a time-specific manner and in our system parallel *ZEB2* expression, then intervene to occupy the enhancers and cause or contribute to negative regulation of *ZEB2*. It is also not excluded that ZEB2 and HOXB2 (and SOX10) co-control each other both by feedback and feedforward regulations, which remains to be investigated in our iPSCs. Further studies are required to identify other TFs acting as negative and/or positive regulators of the neural enhancers we have identified. Interestingly, the expression levels of the other genes located in our *ZEB2* region of interest do not change significantly during neural differentiation, suggesting that of the many genes that flank the gene desert, only *ZEB2*—as the encoded TF is crucial for proper embryonic development—needs precise and dynamic regulation.

Of the whole spectrum of mono-allelic mutations described in MOWS patients, 20% are composed of large gene deletions (including cytogenetically detectable deletions) that very often are described to affect a significant part or the entire *ZEB2*, but sometimes also neighboring, such as downstream located genes (22). Such patients have very severe phenotypes that encompass various, classical MOWS defects, but could very well have other associated defects due to loss of function of (one or more of) the other genes. Recently, a patient without a mutation in the *ZEB2* protein-coding exon sequences, but with clear MOWS, was identified (71). Sequencing identified a 69 kb-long duplication, located in chr2:145218807–145287401, containing exons 1 and 2, as well as intron 1 and (part of) intron 2 of *ZEB2* (73). In a screening for copy number variants in genes related to Hirschsprung Disease, four patients have been described with *ZEB2* duplications of part of exon 1 and all of exon 2, ranging from 1.42 to 1.99 kb (72). Of these patients, three presented mutations in the *RET* gene and, in two of these three, also a *SOX2* duplication. In line with our results obtained here, these duplications might hamper the formation of the TAD structure we identified here and therefore have an effect on *ZEB2* transcription, including blocking (candidate) TF-binding promoted DNA looping needed for *ZEB2* upregulation.

We also found a sub-TAD formed between the whole *ZEB2* gene and the 5′-downstream gene *GTDC1* (glycosyltransferase-like domain-containing 1). In 2017, Aksoy and co-workers described the sequencing, via DNA paired-end tag, of a patient affected by global developmental delay, language impairments and intellectual disability (73). In this patient, they found a *de novo t*(2;8) translocation affecting *GTDC1* on chromosome 2, while no annotated gene was involved on chromosome 8. On chromosome 2, the breakpoint is located in intron 5 of *GTDC1*. This translocation might therefore result in an impaired TAD formation between *GTDC1* and *ZEB2*, which might affect the expression of both genes.

Our work clearly indicates that T2C-based functional studies and the identification of novel REs would be beneficial not only for a better understanding of the connection between RE-containing developmental and disease loci, such as of *ZEB2*, and genome structural organization, but also to clinical geneticists who will systematically intensify gene desert sequencing on top of exon-sequencing in diagnostics in the future, and analyze REs in cell-based functional studies.

**Table 1:** Antibodies used in this study

Antibody	Commercial source	Cat. No.	Dilution
OCT4	Abcam	Ab19857	1:250
NCAM1/CD56	R&D systems	AF2408-SP	1:200
SOX2	Immune systems	GT15098	1:400
NESTIN	Biolegend	839801	1:200
PAX6	Biolegend	901301	1:200
TUJ1	Biolegend	801202	1:500
ZEB2	SantaCruz	sc-48789	1:100
Alexa Fluor Donkey α-Rabbit 488	ThermoFisher Scientific	A32790	1:500
Alexa Fluor Donkey α-Goat 594	ThermoFisher Scientific	A32758	1:500
Alexa Fluor Goat α-Mouse 488	ThermoFisher Scientific	A11001	1:500
Donkey α-Rabbit Cy5	Jackson ImmunoResearch	711-175-152	1:500

## Materials and Methods

### Induced pluripotent stem cells

The WTC iPSC line was obtained from Dr Bruce Conklin (The J. David Gladstone Institutes, San Francisco, CA, USA). These cells were cultured feeder-cells free on 6-well-plates coated with 1% Geltrex in Essential-8 (E8) Basal Medium (ThermoFisher Scientific), and the medium was changed daily. For their neural induction, a modified version of the protocol by Singec and co-workers was used (74). In brief, 70%-confluent cells were changed to Neural Induction Medium (NIM): DMEM/F12, supplemented with 20% knock-out serum replacement, 1 mm non-essential amino acids and 0.1 mm β-EtSH (all from ThermoFisher Scientific), and a mix of inhibitors (abbreviated as DAP), i.e. 2 μM dorsomorphin (BMP-inhibitor, Tocris Bioscience), 2 μM A83–01 (TGFβ-inhibitor, Tocris Bioscience) and 2 μM PNU-74654 (WNT-inhibitor, Sigma). After 6 days (D6) the medium was replaced with neural maturation medium: DMEM/F12, 1 mm non-essential amino acids, 0.1 mm β-EtSH, 1× N2 and 1× B27 without vitamin A (both ThermoFisher Scientific), and the medium was changed every day until D15 (NPC-state). A schematic overview of the culturing conditions and differentiation protocol is depicted in Figure 1A.

### RNA extraction and RNA-sequencing

RNA was extracted using TRIZOL (Sigma) according to the manufacturer’s instruction, and purified using standard extraction and purification by phenol:chloroform and precipitation by isopropanol.

Total RNA for triplicates of three timepoints was checked for quality on an Agilent Technologies 2100 Bioanalyzer using a RNA nano assay. All samples had an RIN value >9.10. Triplicate RNA-Seq libraries were prepared according to the Illumina TruSeq stranded mRNA protocol (www.illumina.com). Briefly, 200 ng of total RNA was purified using poly-T oligo-attached magnetic beads to end up with poly-A containing mRNA. The poly-A tailed mRNA was fragmented, and cDNA was synthesized using SuperScript II and random primers in the presence of Actinomycin D. The cDNA fragments were end repaired, purified with AMPure XP beads, A-tailed using Klenow exo-enzyme in the presence of dATP. Paired end adapters with dual index (Illumina) were ligated to the A-tailed cDNA fragments and purified using AMPure XP beads. The resulting adapter-modified cDNA fragments were enriched by PCR using Phusion polymerase as follows: 30 s at 98°C, 15 cycles of (10 s at 98°C, 30 s at 60°C, 30 s at 72°C) and 5 min at 72°C. PCR products were purified using AMPure XP beads and eluted in 30 μL of resuspension buffer. One microliter was loaded on an Agilent Technologies 2100 Bioanalyzer using a DNA 1000 assay to determine the library concentration and to check the quality. Cluster generation was performed according to the Illumina TruSeq SR Rapid Cluster kit v2 (cBot) Reagents Preparation Guide (www.illumina.com). Briefly, 18 RNA-Seq libraries were pooled together to get a stock of 10 nM. One microliter of the 10 nM stock was denatured with NaOH, diluted to 6 pm and hybridized onto the flowcell. The hybridized products were sequentially amplified, linearized and end-blocked according to the Illumina Single Read Multiplex Sequencing user guide. After hybridization of the sequencing primer, sequencing-by-synthesis was performed using the HiSeq 2500 with a single read 50-cycle protocol followed by dual index sequencing.

Illumina reads were mapped against the GRCh38 human reference using HiSat2 (75). Gene expression values were called using htseq-count (version 0.11.2) and Ensemble release 96 and transcript annotation (76). Sample QC and differential expression analysis have been performed in R environment for statistical computing (version 3.6.2, https://www.R-project.org/), using DESeq2 (version 1.20.0;) with the ashr log fold shrinkage methodology (http://bioconductor.org/packages/release/bioc/html/DESeq2.html) and tidyverse (version 1.3.0; https://github.com/tidyverse/tidyverse) (77–79).

### Indirect immunofluorescence

hiPCs were plated on Geltrex^®^ coated chamber slides. When confluent, the cells were differentiated as described before on the slides and harvested on the selected time points (D0, D6 and D15). The cells were washed three times with PBS (Sigma) and fixed for 15 min at room temperature (RT) with 4% paraformaldehyde (PFA) and washed again thrice 5 min with PBS. The cells were then permeabilized with 100% ice-cold methanol at −20°C for 10 min, washed with PBS thrice for 5 min and then blocked for 1 h at RT in blocking buffer (5% normal goat or donkey serum (Jackson Immunoresearch), 0.3% Triton-X in PBS). Cells were incubated overnight (O/N) at 4°C with the primary antibodies (Table 1) in antibody dilution buffer (1% BSA, 0.3% Triton-X in PBS) in a humidity chamber. The next day, the cells were washed three times with PBS for 5 min and incubated with the corresponding fluorescent secondary antibody in antibody dilution buffer for 1.5 h at RT in the dark. After washing the cells three times for 5 min with PBS, the cells were mounted with Mowiol (Sigma) containing DAPI (1:1000, Sigma-Aldrich) and dried O/N in the dark. Images were acquired with a Leica SP5 confocal microscope.

**Table 2:** shRNA sequences used in KD experiments

shRNA	Target sequence	Oligonucleotide sequence
shRNA HOXB2 #1	CCGCCAAGAAACCCAGCCAAT	CCGG**CCGCCAAGAAACCCAGCCAAT**CTCGAG**ATTGGCTGGGTTTCTTGGCGG**TTTTT
shRNA HOXB2 #2	CGGCCTTTAGCCGTTCGCTTA	CCGG**CGGCCTTTAGCCGTTCGCTTA**CTCGAG**TAAGCGAACGGCTAAAGGCCG**TTTTT
shRNA HOXB2 #3	CTTGGATGAAAGAGAAGAAAT	CCGG**CTTGGATGAAAGAGAAGAAAT**CTCGAG**ATTTCTTCTCTTTCATCCAAG**TTTTT
shRNA SOX10 #1	CCTCATTCTTTGTCTGAGAAA	CCGG**CCTCATTCTTTGTCTGAGAAA**CTCGAG**TTTCTCAGACAAAGAATGAGG**TTTTT
shRNA SOX10 #2	GCAGCCAGTATATACGACACT	CCGG**GCAGCCAGTATATACGACACT**CTCGAG**AGTGTCGTATATACTGGCTGC**TTTTT
shRNA SOX10 #3	GCTGCTGAACGAAAGTGACAA	CCGG**GCTGCTGAACGAAAGTGACAA**CTCGAG**TTGTCACTTTCGTTCAGCAGC**TTTTT
shRNA FOXD2 #1	CTTCTCTATAGACCACATCAT	CCGG**CTTCTCTATAGACCACATCAT**CTCGAG**ATGATGTGGTCTATAGAGAAG**TTTTT
shRNA FOXD2 #2	GCCTTCCTTCTCTATAGACCA	CCGG**GCCTTCCTTCTCTATAGACCA**CTCGAG**TGGTCTATAGAGAAGGAAGGC**TTTTT
shRNA FOXD2 #3	CGAGGCAGACTTAGCCGAGGA	CCGG**CGAGGCAGACTTAGCCGAGGA**CTCGAG**TCCTCGGCTAAGTCTGCCTCG**TTTTT
scrambled control	CAACAAGATGAAGAGCACCAA	CCGG**CAACAAGATGAAGAGCACCAA**CTCGAG**TTGGTGCTCTTCATCTTGTTG**TTTTT

**Table 3:** Primer sequences used for RT-qPCR analyses

Primer	Oligonucleotide sequence
ACTIN_Fwd	TCCCTGGAGAAGAGCTACGA
ACTIN_Rev	AGCACTGTGTTGGCGTACAG
HOXB2_Fwd	GAATTTGAGAGGGAGATTGGGT
HOXB2_Rev	GGGAAGGTTTGCTCGAAAGG
SOX10_Fwd	ACAAGAAAGACCACCCGGAC
SOX10_Rev	AAGTGGGCGCTCTTGTAGTG
FOXD2_Fwd	TGCGCCAAAGCCTTCTAC
FOXD2_Rev	TGGCCCATGATGTGGTCTAT

### Targeted chromatin capture

The T2C protocol was adapted from Kolovos and co-workers (80). Cells were collected at D0, D6 and D15 of neural differentiation, using Accutase^®^ (ThermoFisher Scientific) and passed through a 40-μm cell strainer (BD Falcon). 2.5x10^6^ cells were used for each time point; the cells were cross-linked using 1% formaldehyde at room temperature (RT, 24°C) for 10 min and quenched with 0.125 M glycine. Subsequently, cells were lysed using cold lysis buffer containing 10 mm Tris-HCl pH 8.0, 10 mm NaCl, 0.5% NP-40 and complete protease inhibitors (Roche). Chromatin was digested with the *Apo*I restriction enzyme (New Englad Biolabs) (400 U/sample) overnight in a thermomixer (VWR) at 37°C at 900 rpm. The digested products were purified via Phenol:Chloroform. The diluted DNA fragments were ligated with T4 DNA Ligase High Concentration (100 U/sample; ThermoFisher Scientific) overnight at 16°C, and then 30 μL of 10 mg Proteinase K/ml (300 μg) were added and incubated at 65°C for 4 h, followed by 30 μL of 10 mg RNAse A/mL (300 μg) for 1 h at 37°C, before proceeding to further purification with Phenol:Chloroform. In total, 6 μg of the resulting chromatin were then linearized using the frequent 4 bp-cutting enzyme *Dpn*II (New England Biolabs) (1 U/μg of DNA) overnight at 37°C, while shaking in a thermomixer at 400 rpm. The day after, the material was precipitated by sodium acetate/ethanol before proceeding to T2C library preparation.

For each sample, a T2C Library was prepared using 250 ng of linearized chromatin. The samples were re-buffered to 10 mm Tris-HCl, pH 8 by a standard AMPure XP (Agencourt) bead clean-up procedure. The chromatin was sheared to 300–400-bp fragments by a S220 Covaris (Covaris Inc.). Concentration was determined by Quant-it high sensitivity (ThermoFisher Scientific). For each sample 100 ng of sheared chromatin was end-repaired and A-tailed using the Kapa hyper prep kit (Roche) according to the manufacturer’s instructions. SeqCap library adaptors were ligated followed by AMPure bead clean-up. The pre-capture library was amplified by PCR using KAPA HiFi hotstart readymix for 9 cycles. The amplified pre-capture library was purified by bead clean-up and quantified by Bioanalyzer DNA1000 assay (Agilent) according to the manufacturer’s instructions. A *ZEB2* hg19-based design was ordered at NimbleGen (Roche) with baits located between 143270465 and 150642631 of chromosome 2. A hybridization mixture per sample was prepared with 2 μg pre-capture library, 1 mm HE-index-oligo, 1 mm HE universal oligo, COT Human DNA, AMPure XP reagent and added to 4.5 μL of pre-ordered baits and subsequently hybridized for 16 h at 47°C. Post hybridization, the samples were washed according to the instructions in the Nimblegen SeqcapEZ Hypercap workflow (Roche), the chromatin captured using capture beads. The captured library was amplified by PCR using Kapa HiFi mix and purified by AMPure XP beads. The captured library was quantified by Nanodrop spectrophotometer and the quality was assessed using a Bioanalyzer DNA1000 assay. Finally, the captured T2C libraries were denatured and sequenced on an Illumina HiSeq2500 sequencer as described for RNA-seq but with a custom recipe of 6 dark cycles, followed by paired end 101 sequencing with single index using the rapid v2 chemistry according to manufacturer’s instructions (Illumina).

T2C analysis was performed as described in Kolovos and co-workers (80). In short, reads were aligned to the human GRCh37 reference genome with the BWA aligner and the BWA-backtrack method. Alignments were subsequently annotated with the restriction fragments in which they were located. The proximity matrix was then constructed from the mapped primary alignments with their mapped primary mates. Further analyses and filtering based on the proximity matrix was performed in the R environment for statistical computing.

### Cloning and luciferase assay and shRNA experiments

Clonable DNA fragments encoding the candidate putative enhancers and the minimal promoter of human *ZEB2* were produced as a single gBlock (IDT), which was then inserted in the luciferase-based pGL4.10 vector (Promega). HEK293T cells, cultured in high glucose (4.5 g/L) DMEM supplemented with 10% FBS, were co-transfected with 1.5 μg of luciferase construct, containing different combinations of enhancers with the ZEB2 minimal promoter, and 50 ng Renilla-based vector, using Lipofectamine-2000 in a 1:1.5 ratio. Empty pGL4.10 was used as negative control. NPCs were transfected with Amaxa Nucleofector II, using the Kit V (Lonza) and transfection program A-33. 4.5 μg of luciferase-construct were transfected with 50 ng of Renilla-encoding vector to 400 000 cells grown in a 12-well plate. After 24 h the cells were lysed in 1x Passive Lysis Buffer (PLB) (Promega). Luciferase and Renilla activity were measured in a Varioskan Lux Microplate reader (ThermoFisher Scientific) using the Dual-Luciferase Reporter Assay System (Promega). Enhancer activity was calculated as the fold-change of Luciferase normalized to Renilla activity. Each transfection was performed three times, and of each transfection, three technical replicates were measured**.**

For the KD experiments, shRNAs for *HOXB2*, *SOX10* or *FOXD2* were transfected/co-transfected with the luciferase-constructs. Table 2 lists the shRNA sequences used for the KD. Medium was refreshed 24 h after transfection, and the cells were harvested 48 h after the transfection. To address the KD efficiency, RNA was isolated, and cDNA was synthesized as described above, and expression levels were tested by real-time quantitative PCR (RT-qPCR). RT-qPCR was performed using SybrGreen dye (BioRad) on a CFX96 T1000 thermal cycler. All data shown are averages of three independent biological replicates and three technical replicates, normalized to ß-ACTIN. Primers are listed in Table 3. Luciferase activity after KD was performed as described above.

## Data Availability

T2C data are available under the GEO accession number GSE147000.

## Supplementary Material

Supplementary_File_1_ddaa141

Supplementary_File_2_ddaa141

Supplementary_Files_ddaa141
