# Supplementary material for: Targeted chromatin conformation analysis identifies novel distal neural enhancers of ZEB2 in pluripotent stem cell differentiation
Source: Hum Mol Genet. 2020 Jul 6;29(15):2535–50. doi: 10.1093/hmg/ddaa141 (PMC7471508; doi:10.1093/hmg/ddaa141)
Supplement: Supplementary_Files_ddaa141 [file supplementary_files_ddaa141.pdf]

Birkhoff JC *et al.* – Supplementary Figure Legends and Files

Targeted Chromatin Conformation (T2C) analysis identifies novel distal neural enhancers of *ZEB2* in pluripotent stem cell differentiation.

Supplementary Figure 1. Schematic overview of published enhancers and chromatin architecture changes and overview of T2C Region of Interest (ROI) in this study.

A) Known identified *ZEB2* intragenic enhancers as described in Bar-Yaacov et al (31) and of predicted looping between SNPs located in *TEX41* (a non-ccoding RNA) and *ZEB2* (34). B) T2C ROI considered for this study with protein-encoding genes (black boxes) and non-coding RNAs (orange boxes). < or > indicates the direction of transcription.

Supplementary Figure 2. Normalized steady-state RNA values of *ZEB2* and selected marker genes at the considered time points of neural differentiation.

The same genes considered for differential expression in Figure 1 (see main text) are assessed here for their temporal dynamics.

Supplementary Figure 3. Distribution and frequency of fragments generated by *Apo*I in the T2C region of interest and T2C quality controls.

A) Density plot of fragment distribution according to the size of the obtained fragments. B) fragment counts versus the size of the individual fragments generated by *Apo*I. C) Number of *cis* and *trans* targeted read-pairs in the proximity matrix. D) Proportion of targeted interaction over 1 kb apart, as compared with their score in read pairs. E) Cumulative number of read-pairs versus the distance of binned (per 50 kb) targeted interactions.

Supplementary Figure 4. *Apo*I fragments-based high resolution T2C maps of *ZEB2* gene chromatin dynamics during differentiation.

T2C maps can be reconstructed at *Apo*I fragments high resolution for local regions of ~100-150kb as in the case of *ZEB2* (136kb) confirming the existence of several intragenic loopings.

Supplementary Figure 5. mRNA expression of annotated genes and long non-coding RNAs located in the considered T2C region of interest (ROI).

A) Normalized values and log2 Fold Change heatmap of genes. B) non-coding RNAs located in the T2C ROI.

Supplementary Figure 6. mRNA expression of transcription factors for which a consensus motif has been identified in the newly identified enhancers.

Supplementary File 1. Coordinates of all *Apo*I fragments interactions for the considered T2C region of interest.

Supplementary File 2. Coordinates of all proximity interactions as obtained from the 20-kb maps (in main text Figure 3) for the three TADs.

A

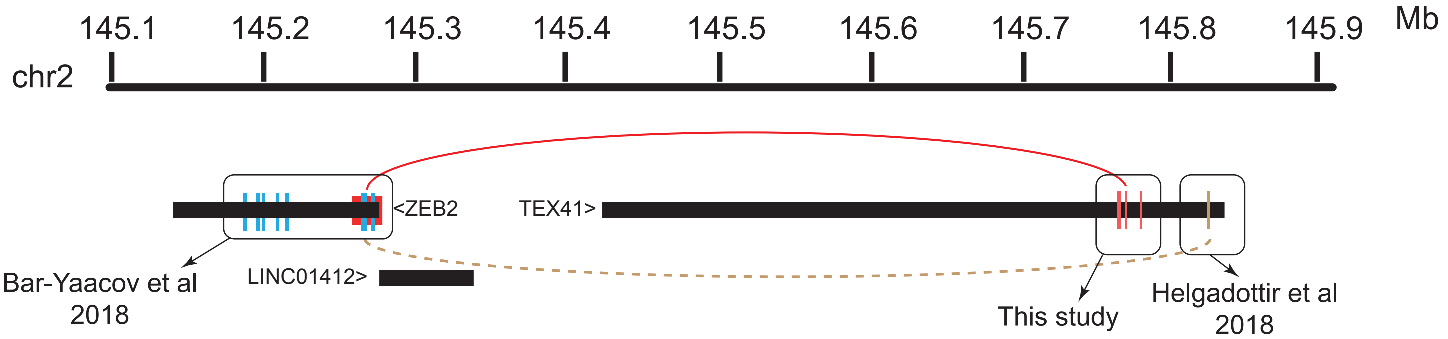

B

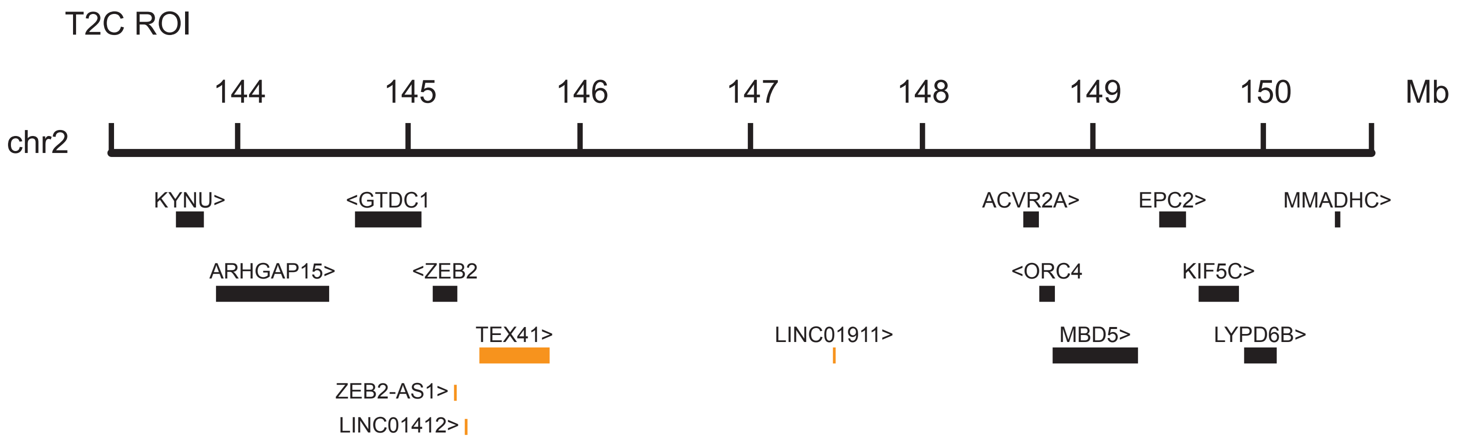

Birkhoff et al 2020 - Dynamics of ZEB2 locus during neural differentiation.

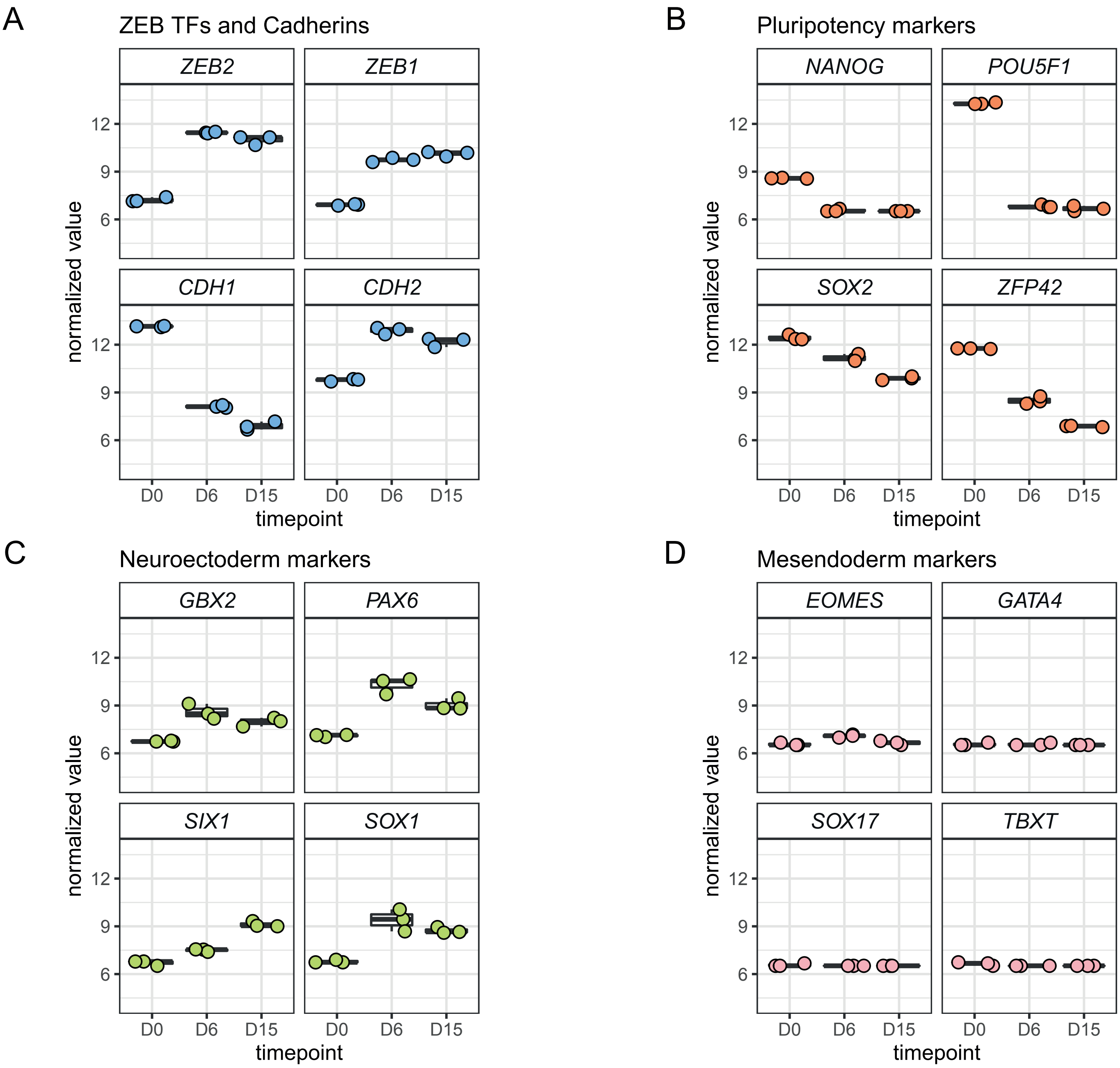

Birkhoff et al 2020 - Dynamics of ZEB2 locus during neural differentiation.

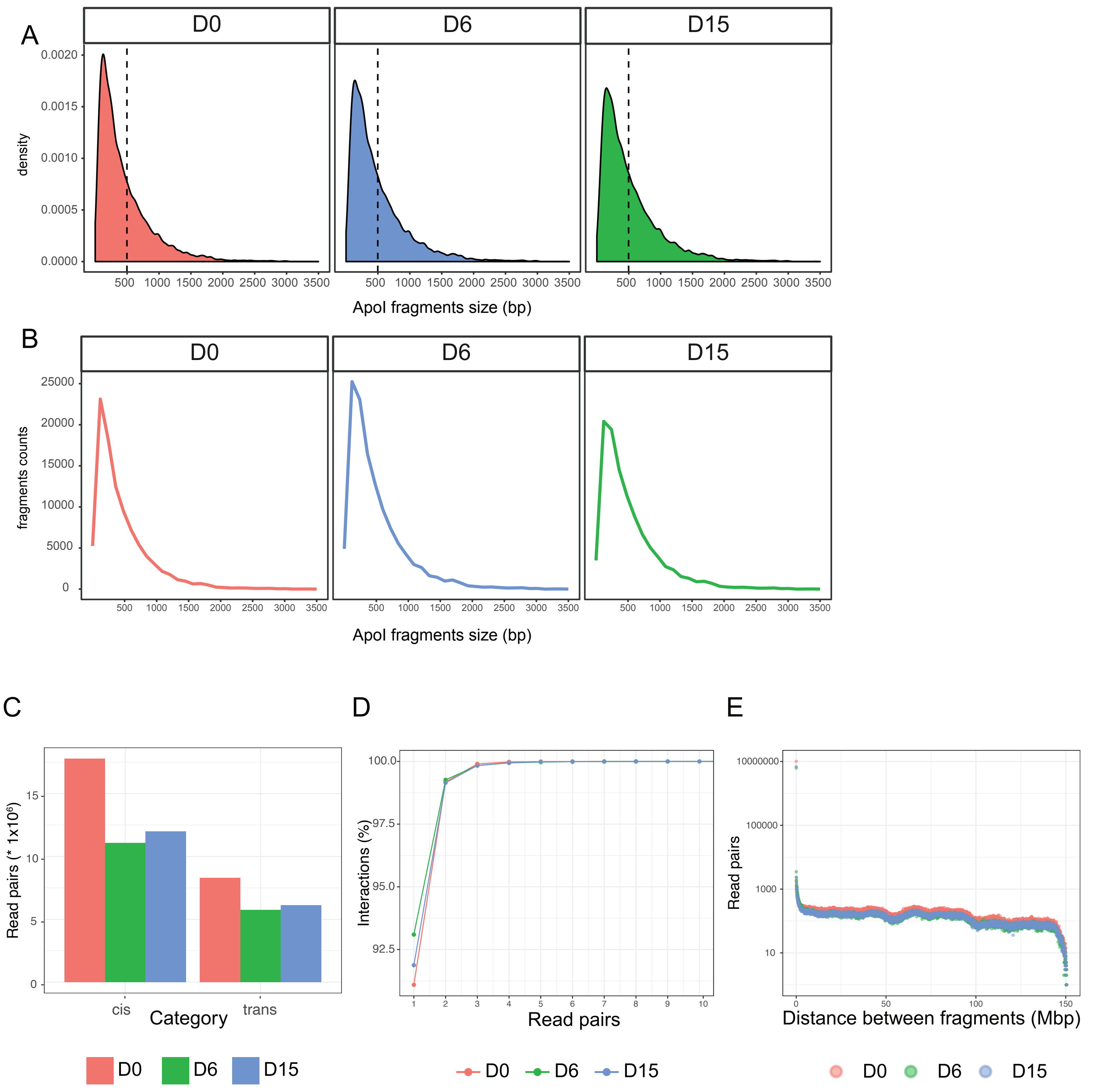

Birkhoff et al 2020 - Dynamics of ZEB2 locus during neural differentiation.

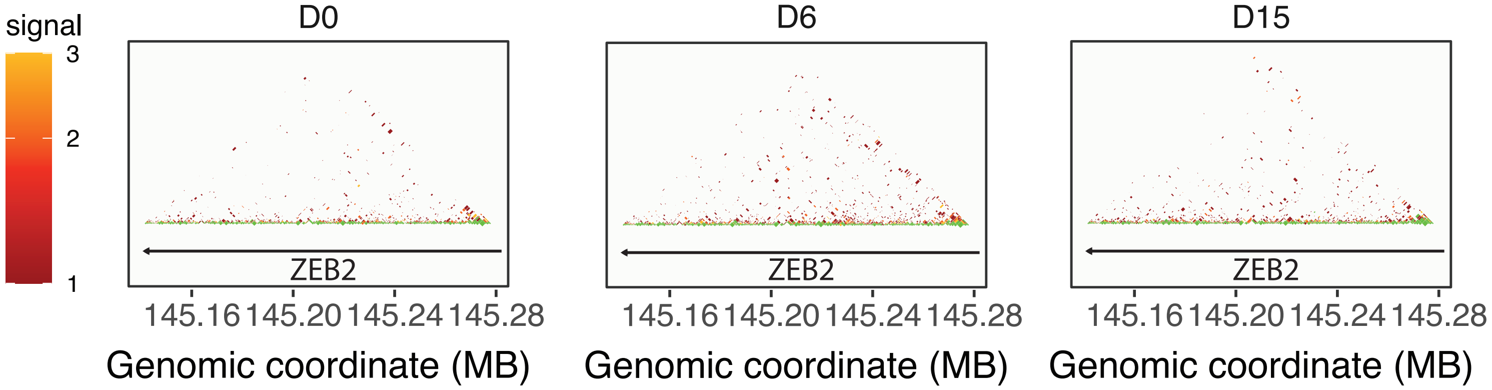
signal
3
2
1
D0
D6
D15
ZEB2
ZEB2
ZEB2
145.16 145.20 145.24 145.28
145.16 145.20 145.24 145.28
145.16 145.20 145.24 145.28
Genomic coordinate (MB)
Genomic coordinate (MB)
Genomic coordinate (MB)

Supplementary Figure 5

A

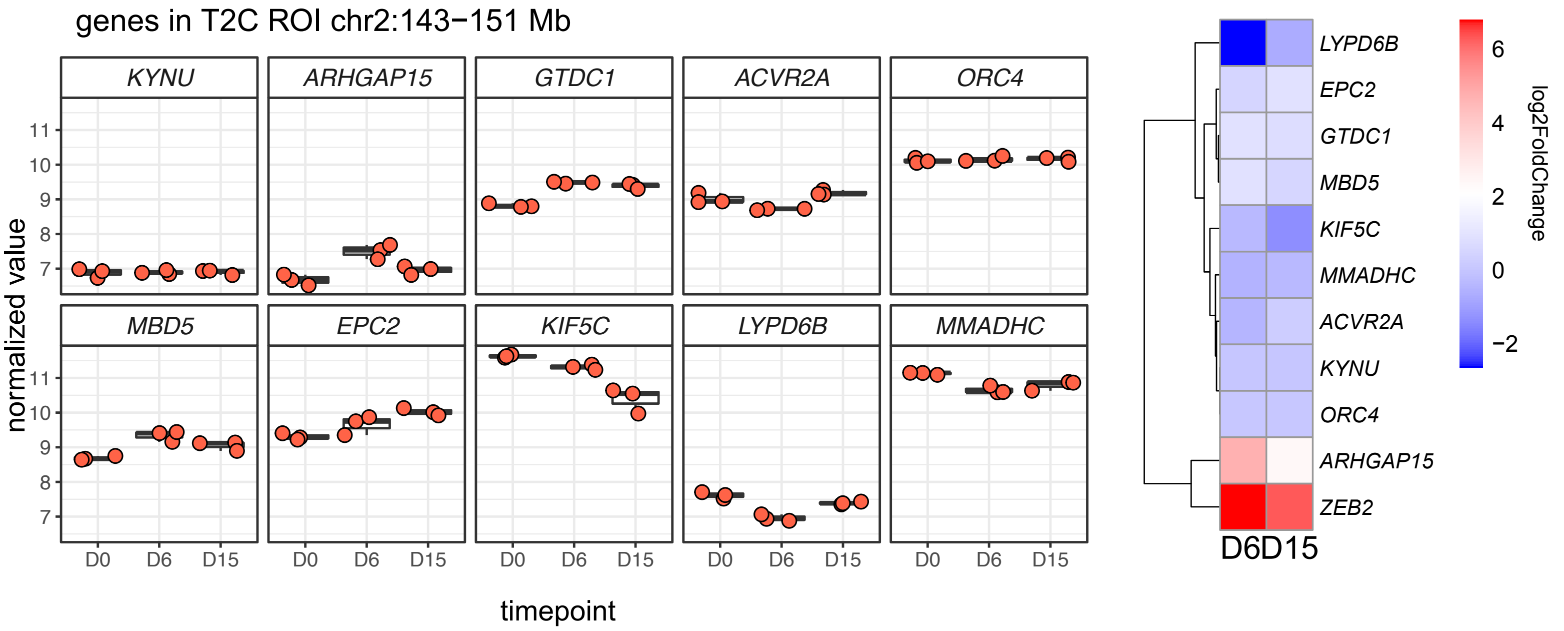

B

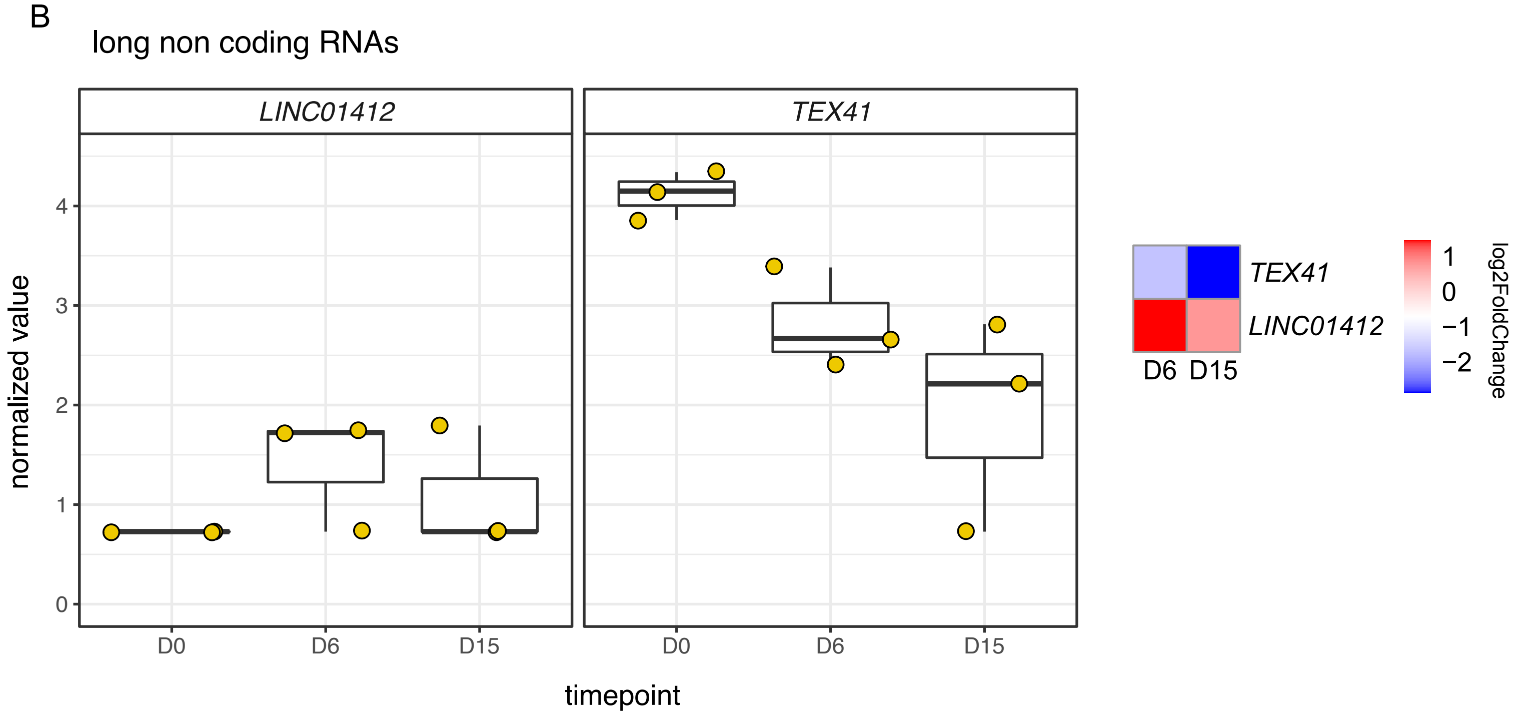

Birkhoff et al 2020 - Dynamics of ZEB2 locus during neural differentiation.

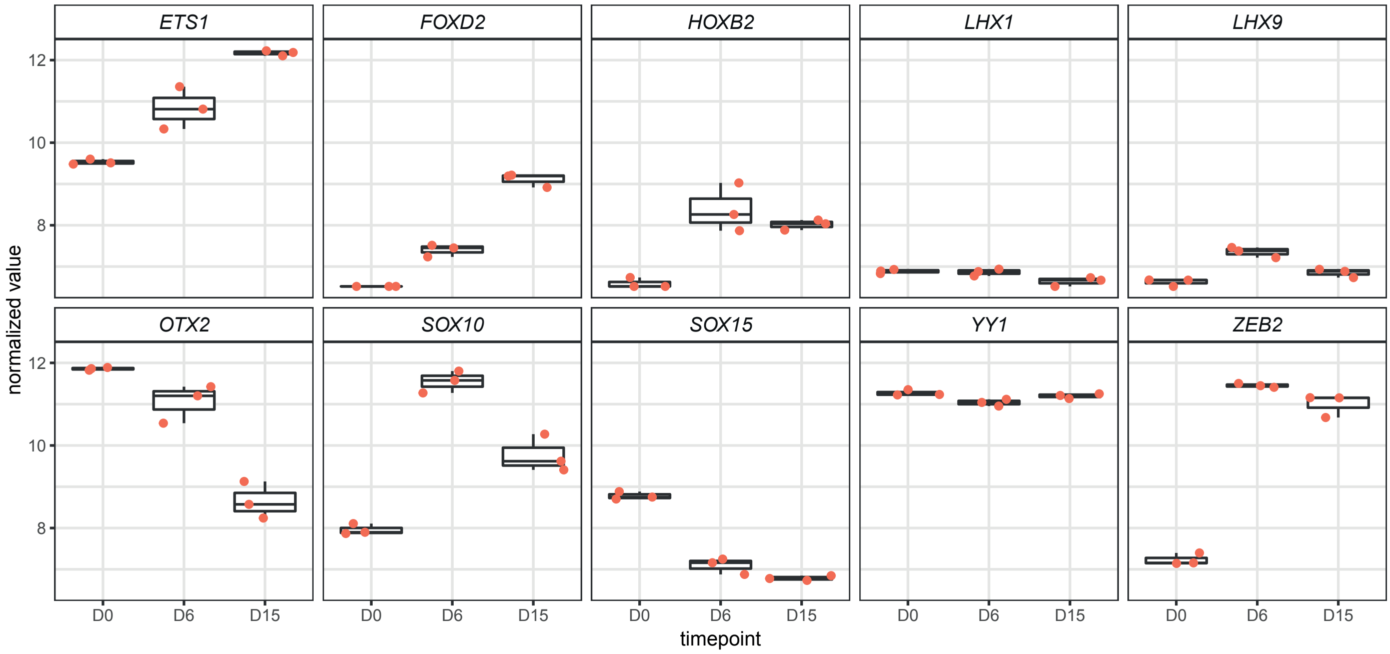

Birkhoff et al 2020 - Dynamics of ZEB2 locus during neural differentiation.
